# In-Cabin Monitoring System for Autonomous Vehicles

**DOI:** 10.3390/s22124360

**Published:** 2022-06-08

**Authors:** Ashutosh Mishra, Sangho Lee, Dohyun Kim, Shiho Kim

**Affiliations:** School of Integrated Technology, Yonsei University, Incheon 21983, Korea; ashutoshmishra@yonsei.ac.kr (A.M.); sangholee@yonsei.ac.kr (S.L.); kimdh5032@yonsei.ac.kr (D.K.)

**Keywords:** artificial intelligence, high-level autonomous driving, in-cabin monitoring, intelligent camera, irregular situations

## Abstract

In this paper, we have demonstrated a robust in-cabin monitoring system (IMS) for safety, security, surveillance, and monitoring, including privacy concerns for personal and shared autonomous vehicles (AVs). It consists of a set of monitoring cameras and an onboard device (OBD) equipped with artificial intelligence (AI). Hereafter, this combination of a camera and an OBD is referred to as the AI camera. We have investigated the issues for mobility services in higher levels of autonomous driving, what needs to be monitored, how to monitor, etc. Our proposed IMS is an on-device AI system that indigenously has improved the privacy of the users. Furthermore, we have enlisted the essential actions to be considered in an IMS and developed an appropriate database (DB). Our DB consists of multifaced scenarios important for monitoring the in-cabin of the higher-level AVs. Moreover, we have compared popular AI models applied for object and occupant recognition. In addition, our DB is available on request to support the research on the development of seamless monitoring of the in-cabin higher levels of autonomous driving for the assurance of safety and security.

## 1. Introduction

The Society of Automotive Engineers (SAE) and the National Highway Traffic Safety Administration (NHTSA) have suggested a level of autonomy in autonomous vehicles (AVs) from level 0 (no autonomy) to level 5 (full autonomy) [1,2,3]. Vehicles at level 4 and beyond are supposed to perform all driving tasks. According to NHTSA, a level 5 AV (i.e., fully autonomous vehicle (FAV)) is an automated driving system that can perform all the driving in all circumstances. Every occupant (human) is just a passenger and is never required to be involved in driving [3]. There is no one responsible in charge, and all the occupants are passengers. They are free from driving and vehicle control responsibilities. All occupants are free to perform other tasks of their interests, including relaxing during their commute. What should be the appropriate position of the camera for the in-cabin of a higher level of autonomous driving?

**Figure 1:**
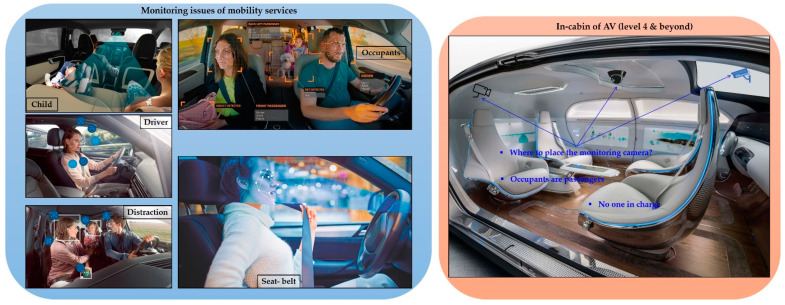
An illustration of the monitoring issues of mobility services and in-cabin of a level 4 and beyond AV.

Figure 1 elicits the need for in-cabin monitoring and the problem associated with the in-cabin monitoring. It also depicts the problem of the installation of monitoring camera(s) for the in-cabins of high levels of autonomous driving (e.g., level 4 and beyond AVs). The high levels of autonomous driving reduce the risk of fatality and the stress of daily commute driving. In addition, it provides extra time, luxury, and stress-free transportation experiences to the occupants in their daily commute [4,5]. FAVs (level 5 AV) can be especially suitable for the modern commute and allow occupants to perform other important tasks, enjoy luxury, rest, and so on. It removes the necessity of the person in charge inside the vehicle. However, it enforces an inevitable necessity of the monitoring system inside the cabin. In addition, the FAV has also considered a solution for increasing demand for public and shared vehicles (such as ridesharing, carsharing, and car-full services). Interestingly, passengers in such shared public vehicles will be unknown to others. An in-charge plays an important role in monitoring and safety of the passengers (occupants) inside a vehicle. Any discomfort, vandalism, or irregular situation in the cabin of the vehicle is supposed to be addressed and resolved by the in-charge of the vehicle.

**Figure 2:**
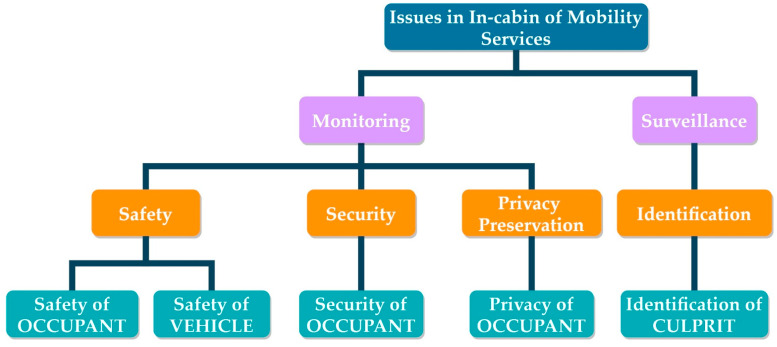
Monitoring and surveillance are essential in highly automated driving. Broader categorizations are related to the safety, security, and privacy of the occupants.

The absence of a vehicle in-charge requires a robust solution to ensure the security and safety of all occupants. Especially in level 5 AVs, in-cabin monitoring is of utmost importance. Furthermore, the vehicle itself requires protection from any malicious behavior by the occupants. Therefore, multi-pronged in-cabin monitoring of level 4 and beyond AVs in real-time is crucial [5,6]. Plenty of past works have reported in-cabin monitoring for different purposes However, in-cabin monitoring in level 4 and beyond AVs should intend for the safety and security of the occupants and the vehicle [5,6]. The safety of each occupant implies their physical protection. On the other hand, the security of occupants indicates their information protection. Moreover, the safety of a vehicle is meant for protection from its misuse, damage, and exploitation. Issues of in-cabin monitoring in automated mobilities are broadly categorized as below (in Figure 2):

Monitoring occupant’s safety represents the assurance of no harm to occupants such as their bodily protection (no body parts outside, occupant’s seating, and other comforts, food or beverages spilling, etc.), ease of commute (motion sickness, dizziness, etc.), ease of vehicle–occupant interaction (infotainment-related, information--related, alert- or alarm-related, etc.), and so on. Whereas vehicle safety monitoring involves the safety of vehicle hardware components, including vehicle controls, cleanness inside, and energy-efficient operations for saving energy. Furthermore, monitoring the security of the occupants deals with the mitigation of vandalism, teasing, abuse, etc. Therefore, there must be:

1. Appropriate monitoring perspectives for level 4 and beyond AVs;
2. Sensing capabilities for the aforementioned issues;
3. A significant dataset is another bottleneck of such an in-cabin monitoring system:

In this paper, we have deduced an intelligent in-cabin monitoring system (IMS) that can perform the perception and sensing of irregular situations in level 4 and beyond AVs. Our main contributions to this research are listed below:

1. The installation of an AI camera for efficient in-cabin monitoring in level 4 and beyond AVs;
2. Enlisting the crucial task of in-cabin monitoring in level 4 and beyond AVs;
3. The creation of a suitable dataset for the development of an AI camera;
4. Occupants and object recognition in the cabin of level 4 and beyond AVs;
5. Privacy-preserved monitoring in the public domain.

We have created our dataset to develop an intelligent IMS. The proposed system has a single camera assisted with artificial intelligence (AI) to monitor the in-cabin of level 4 and beyond AVs. This study suggests an efficient method of in-cabin monitoring by providing a single-shot intelligent monitoring approach. Furthermore, it provides the basis and requirements of an IMS for objects and occupant detection under irregular situations considering privacy concerns. Privacy is another crucial hurdle in such public monitoring systems. There must be the preservation of privacy to prevent personal information from being misused. Blurring or masking faces while monitoring causes difficulties in further monitoring tasks such as intent, emotions, etc. Therefore, some significant way out is essential for maintaining privacy in such monitoring systems.

## 2. In-Cabin Monitoring

Previous researchers have reported several works incorporating in-cabin monitoring. Bosch et al. have suggested in-car emotion recognition using eye-tracking and visible light cameras [7]. The authors have reviewed multiple works involving the detection of various emotions, such as anger, fear, depressed feelings, curiosity, embarrassment, urgency, boredom, frustration, etc. In level 4 AVs, driver monitoring is an essential part of the advanced driver assistance systems (ADAS). In [8], the authors have surveyed the issues and trends in connected cars. They have surveyed different works for driver monitoring. Rong et al. have surveyed AI methods used to ensure the in-cabin safety of AVs [9]. They have enlisted popular AI methods used for three different tasks, viz., driver status monitoring, driver assistance, and takeover readiness. Driver status monitoring includes emotion, fatigue, distraction, and attention detection. Driver assistance has intention analysis and traffic hazards warning, and takeover readiness has takeover readiness evaluation. In their survey, they have incorporated multiple works involving AI for these tasks. The driving monitoring and assistance systems have been reviewed in [10]. They studied the driving process considering driver, vehicle, and driving environment. Driver’s distraction, fatigue, and aggressive driving style are focused on in this review.

In [11], an experiment was performed using in-cabin monitoring to study the driver’s behavior and their interaction with automated driving. The aim of this study was to implement a similar approach in FAVs. In-vehicle violence detection is reviewed in [12]. Authors have surveyed multiple articles and AI methods for in-vehicle emotion detection to develop violence detection in carpooling services. In-cabin passenger anomaly detection was suggested in [13]. An evaluation of the in-vehicle monitoring system (IVMS) has been carried out in [14] to reduce risky driving behaviors. An idea of monitoring a vehicle cabin was patented in [15]. They considered a modern vehicle scenario and described their cabin monitoring system as equipped with a camera and a computer-based system. It was supposed to perform detection of the vehicle seat, occupants, their orientation, and seat orientation. A safety and cleaning issue of the in-cabin has been considered in [16] to avoid any malfunctioning or blockage of vehicle controls due to a foreign object such as a bottle, can, electronic devices, keys, books, etc. Likewise, multiple pieces of literature and research have been reported in past for monitoring a vehicle’s cabin. However, an intelligent IMS is still in demand for a reliable implementation of level 4 and beyond AVs in the real world. In high levels of autonomous driving, the aspect of safety and mitigating dangerous situations in case of a crash becomes crucial. Because of the constraints and limitations inside the cabin of the vehicle, Ribas et al. found a challenge in deciding the best sensor positioning [17]. They introduced synthetic data to test the AI models for estimation of occupant’s pose in-cabin. To create photorealistic scenes, they used *MakeHuman*, *Blender*, *Openpose*, and *mask R-CNN* models to build the virtual in-cabin environments containing selected occupants’ postures. However, their simulated dataset has front-facing scenes. They have mainly focused on occupants’ safety in the case of a crash and generated the synthetic dataset containing scenes to fulfill the specifications of European safety regulations. Recently, multiple industries and startups have started working on in-cabin monitoring solutions [18,19,20,21,22,23,24,25,26,27,28,29,30,31,32,33]. However, in-cabin monitoring datasets and robust IMSs for high levels of autonomous driving are still required.

## 3. Safety and Security in Level 4 and beyond AVs

**Figure 3:**
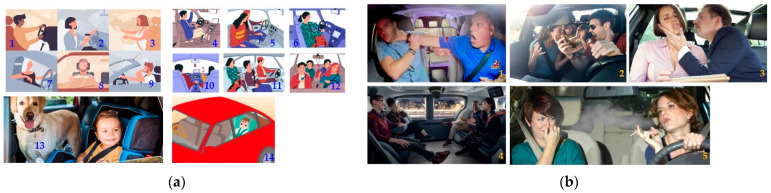
An illustration of (**a**) the safety (for example, no seatbelt: 1, 5, 11; drinking and drive: 2; drowsy and driving: 3; body part outside the vehicle: 4, 7; distracted driving: 6, 9, 10, 12; tense driving: 8; and unattended child and pet: 13, 14). (**b**) Illustration of the security issues in level 4 and beyond AVs (for example vandalism: 1; violence and argument: 2; harassment: 3; personal privacy breach during monitoring in public: 4; discomforted due to smoking: 5).

The safety of a person or object means protection from danger, risk, or injury. Security refers to the protection of individuals and objects against external threats and criminal activities. An AV should have safety and security protection features. Figure 3 illustrates the example scenarios of both safety and security.

The safety of a person or thing is the assurance of security against some unintended accidents. Likewise, security is the protection from deliberate threats. Occupants need both safety and security while the vehicle demands safety from being mishandled. Table 1 enlists the major concerns related to the safety and security of the occupants and vehicle.

Although we have enlisted multiple issues related to the safety and security of occupants and vehicles in this table. However, other issues may be possible in future FAVs depending upon their usage (i.e., personal vehicle, shared vehicle, or the type of vehicle such as goods carrier, transport vehicle, etc.). Therefore, this list needs to be updated from time-to-time.

## 4. Related Works

To enhance the safety and security inside the vehicle’s cabin, multiple works have been reported [5,6,7,8,9,10,11,12,13,14,15,16,17]. Recently various companies have also reported their in-cabin monitoring systems for improving the safety and security of the occupants inside the vehicle [18,19,20,21,22,23,24,25,26,27,28,29,30,31,32,33]. Poon et al. have performed deep learning models, including versions of YOLO, to monitor the driving behavior and in-vehicle occupants [34]. Rong et al. surveyed the AI methods involved in in-cabin monitoring [9]. According to them, AI has enabled a new range of applications and assistance in the vehicle cabin. A dataset of in-car cabin monitoring for vehicle interior monitoring is published by Katrolia et al. in [35]. They have used a single wide-angle depth camera to collect in-car datasets for various scenarios. They have also produced some synthetic data according to the real scenarios. In [36], a test platform has been introduced for visual in-cabin scene analysis and occupant monitoring functions. A hardware implementation has been performed in [37] to monitor the driver and passengers inside an automobile. Pappalardo et al. have applied the decision tree method to analyze the lane support system in [38]. Othman et al. have presented a driver monitoring system using in-cabin monitoring and deep learning [39]. They have gathered a video and telemetry dataset for driver monitoring. It includes the driver’s head pose, heart rate, behavior, drowsiness, unfastened seat belt, etc. They have collected datasets considering nine different drivers (including males and females) for a total of 36 h of footage. Deng has suggested the importance of vehicle in-cabin sensing in their dissertation [40]. A wideband antenna array has been used in their experiment for both vehicle safety and comfort in conventional and future intelligent vehicles. Kashevnik et al. have introduced threat detection during human–computer interactions in the in-cabin driver monitoring system [41].

## 5. Database

An appropriate dataset is an utmost important aspect of the AI-assisted system. The absence of a suitable database (DB) for in-cabin monitoring of occupants elicits a serious bottleneck in an intelligent IMS.

The list of objectives and behaviors performed in our DB is given below in Table 2. We have considered various scenarios for higher levels of autonomous driving.

**Figure 4:**
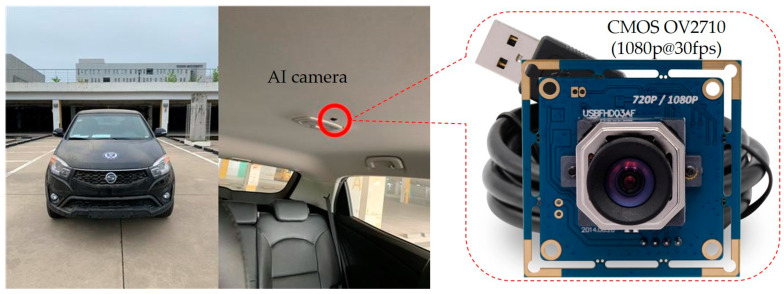
A four-seater vehicle is used in the development of DB. The left image depicts the AV used for DB collection, and the AI camera is discerned on the right side of the image.

We have involved multiple volunteers in the generation of DB and collected data for almost two months in various scenarios. All of them gave their informed consent for inclusion before they participated in this study. Level 4 and beyond AVs require information on the occupancy, occupants, and objects inside the cabin of the AV. Therefore, our DB contains these images corresponding to the desirable scenarios of IMS. For variation, we have collected the DB by considering one occupant, two occupants, three occupants, and four occupants according to their position shown in Figure 4. Our DB has been collected for higher levels of autonomous driving. We have considered level 3 and beyond AVs during our DB generation. Therefore, we have collected the dataset by considering the in-cabins of level 3, level 4, and FAVs.

The in-cabin monitoring DB has a total of 32 videos for different scenarios containing 333,100 frames. These are high-definition videos and images with a resolution of 1280 × 720 pixels. The data acquisition was performed in two sets. In one set of DB, every volunteer was trained to perform actions against the tasks according to the designated scenario. However, in the other set of DBs, the volunteers were not given any prior instructions for performing their tasks. Rather, they were free to perform their action for a given set of tasks. We labeled some of the DB for training the AI camera for object and occupant detection purposes. The label is according to the number of passengers, cigarettes, weapons, cell phones, beverages, and car seat. The rest of the DB was unlabeled for testing the performance of the IMS. Table 3 provides the details of collected images containing selected targets.

### 5.1. Occupants and Objects

**Figure 5:**
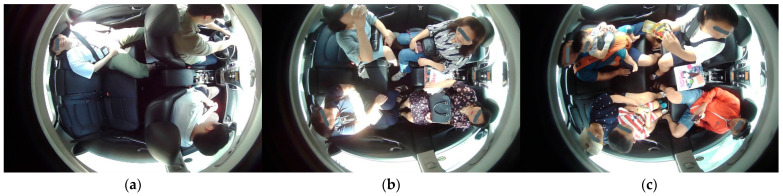
Example images of occupants captured for high levels of autonomous driving: (**a**) Images captured with one action at a time; (**b**) Images captured with multiple actions performed randomly; (**c**) Images of children and objects.

The DB is generated by considering different categories inside the cabin of the AV. These are occupants (passengers), children, animals (pets), smoking items, cellphones, car seat, beverages, bags, belongings, and harmful/dangerous objects. Occupants’ images have been captured for volunteers with one action at a time and performing multiple actions randomly. Figure 5 represents the example images of our DB for occupants.

As the DB has been prepared for higher levels of autonomous driving. We have considered the in-cabin of level 3 AVs to level 5 AVs for DB preparation. Therefore, our DB has an in-cabin dataset for both drivers and occupants. Figure 5a represents the in-cabins of level 3 and level 4 AVs, whereas the in-cabin of a level 5 AV is shown in Figure 5b. We have considered adults, children, and pet animals as possible occupants of AVs. The occupants may or may not have belongings. Accordingly, these images contain possible belonging objects. We have assumed bags, toys, stationery items, etc., as the belongings of the occupants.

### 5.2. Cellular and Electronic Devices

**Figure 6:**
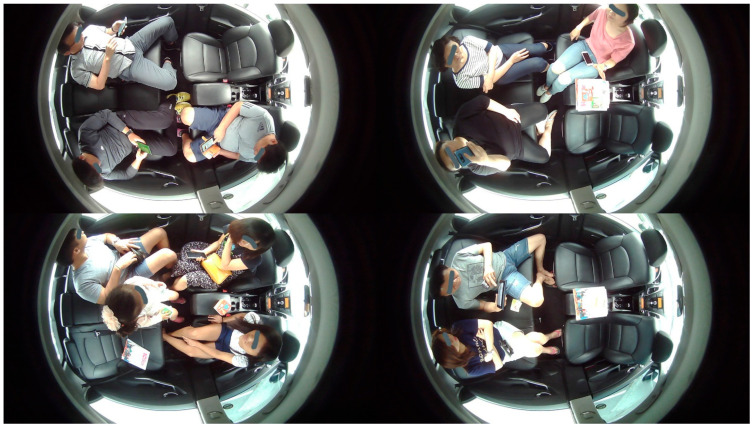
Illustrations are the datasets captured for cellular and electronic devices in the cabin of a vehicle. Here, some of the devices are in use, while others are unused.

There is the possibility of cellular and electronic gadgets inside the cabin of the vehicle. These may be smartphones, laptops, tablet computers, etc. In the case of level 3 and level 4 AVs, they can create driver distractions. However, in level 5 AVs, the detection of such devices is necessary to track a missing or lost device. Figure 6 discerns some images that contain cellular and electronic devices.

### 5.3. Smoking Items

**Figure 7:**
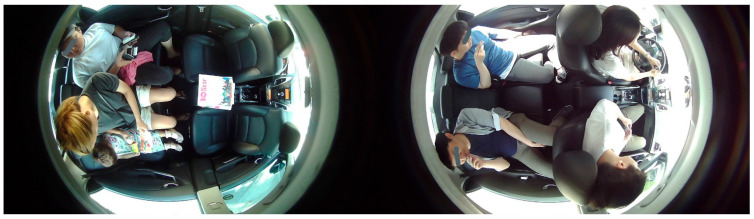

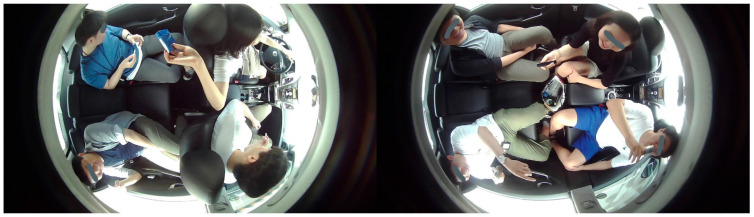
Example images for smoking items inside the cabin of AV.

Smoking is injurious to health and must be avoided in public places. Share or transport vehicles are supposed to be public vehicles. Therefore, there must not be any smoking inside such a vehicle. Hence, the IMS must include smoking item detection for the safety and security of occupants and vehicles. We have considered cigarettes, cigarette lighters, cigarette cases, and e-cigarettes as smoking items (as depicted in Figure 7).

### 5.4. Food and Beverages

**Figure 8:**
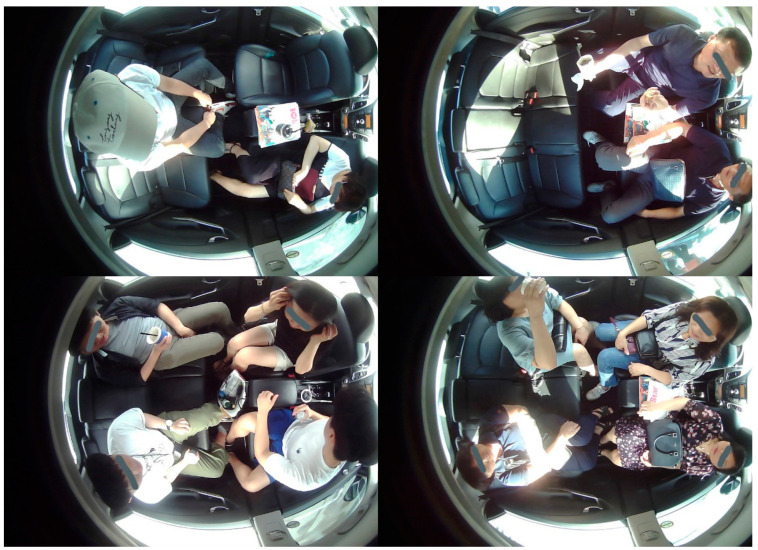
Few images depicting food and beverages in the cabin of a vehicle.

In shared vehicle food and beverages, detection is essential to ensure the safety and cleaning of the in-cabin. During the ride, the eating or drinking of an occupant may disturb other occupants. For example, careless eating or drinking can spill on other people. Furthermore, the leftover litter from food and beverages may cause problems to the occupants using the vehicle afterward. Furthermore, such litter may obstruct vehicle controls and thereby create severity in the safety of the vehicle. Therefore, the IMS should be capable of recognizing bottles, tumblers, coffee, food, beverages, etc., so that it can alarm occupants in case of any inconveniences caused. Figure 8 shows the use of food and beverages in the cabin of a vehicle.

### 5.5. Harmful Objects

**Figure 9:**
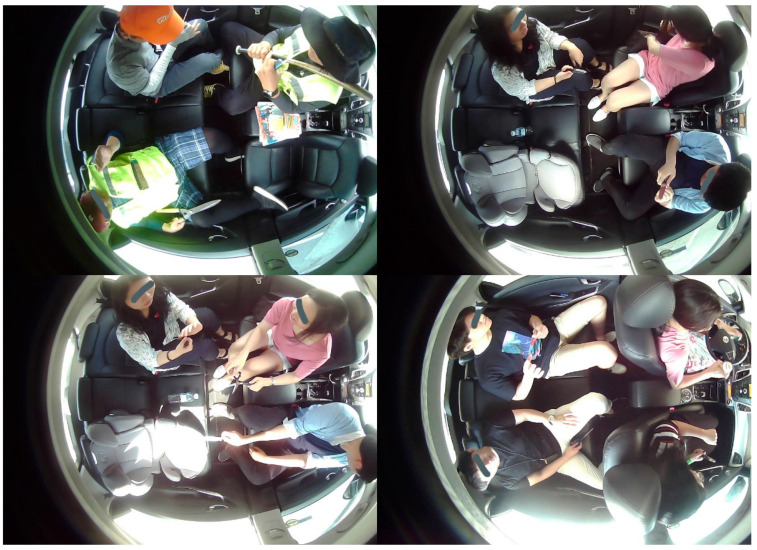
Images for examples of harmful objects in the cabin of an AV.

For ensuring the safety and security in-cabin of higher levels of autonomous driving vehicles, harmful objects, dangerous tools, and weapons must be recognized to avoid and curb any violence or vandalism. We have captured tools in hand such as screwdrivers, scissors, baseball bats, knives, etc., as dangerous objects. A few of such images are showcased in Figure 9.

Therefore, we have considered almost all possible situations inside the cabin of a vehicle and captured images depicting those scenarios in the real world. During dataset collection, we have purposely involved multiple volunteers and captured datasets in different lighting conditions, occupant positions, etc. to incorporate variability in our DB. This variational feature of our DB is helpful in the development of robust AI algorithms for various object detection and recognition purposes.

## 6. Face Anonymization: Privacy-Preserved Monitoring in Public

Privacy is an important concern of public monitoring systems. IMS being a public monitoring system, it also suffers from the same concern. To secure personal information, several countries have imposed severe restrictions on facial recognition techniques in public [42,43,44,45,46,47,48,49,50,51]. Facial anonymization serves to effectively overcome this issue, as the anonymous face protects personal information during in-cabin monitoring. In our previous works, we have proposed an efficient algorithm for face anonymization of the occupants [5,6,52]. The generative adversarial network (GAN) is utilized for facial swapping and reenactment. Face swapping implements the facial transferring from a source face image (a face that is used for replacing the target face) to a target face image (the face that needed to be anonymous). We have recommended using the GAN-generated virtual human faces as the source image. This ensures more reliable anonymization. The preservation of the facial attributes of the target image (such as facial expression, head pose, illumination, background, etc.) is of importance. For proper reenactment, it is recommended to properly superimpose the facial landmarks of both faces. Because perfect anonymization is essential to preserve the intent and emotion on the swapped face, this process usually requires a sequence of GANs to obtain the perfect anonymized face. These GANs perform facial reenactment and segmentation, facial in-painting, and facial blending for realistic facial swapping. This will help in further monitoring tasks such as intent and behavior monitoring using the anonymized face.

**Figure 10:**
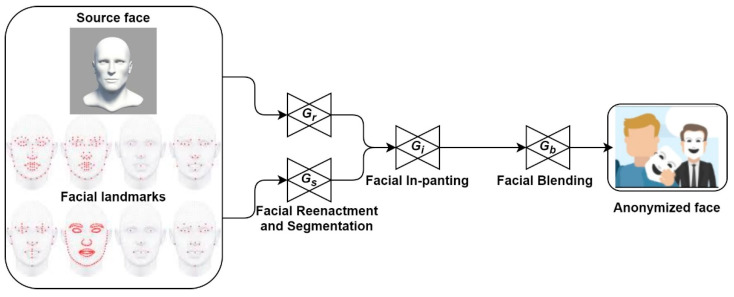
Illustration of facial anonymization for facial privacy preservation. It is required during monitoring of the suspect under an irregular situation.

Figure 10 renders the proposed schema of facial anonymization for facial privacy preservation. Here, G*_r_*, G*_s_*, G*_i_*, and G*_b_* represent the reenactment generator, segmentation generator, in-painting generator, and blending generator, respectively. For proper reenactment, face segmentation and hair segmentation are performed separately for mapping 2D facial landmark positions. A stepwise loss function is used as an objective function. Feature map for the *i^th^* layer is represented as (*ℱ_i_* ∈ ℝ*^C^_i_*^×*H*^*_i_*^×*W*^*_i_*), and the corresponding perceptual loss (*ℓ_perc_*) between image pairs ($I_{1},\;I_{2}$) is:

(1) $$\ell_{\mathrm{perc}}\;(I_{1},\;I_{2})=\sum ((1/C_{i}\times H_{i}\times W_{i})\;\times \;\|{ℱ}_{i}(I_{1})-{ℱ}_{i}(I_{2})\|)$$

The reconstruction loss (*ℓ_rec_*) between pair of images ($I_{1},\;I_{2}$) is (considering every ‘*λ*’ as the corresponding hyperparameter):

(2) $$\ell_{\mathrm{rec}}\;(I_{1},\;I_{2})=\lambda_{\mathrm{perc}}\times \ell_{\mathrm{perc}}\;(I_{1},\;I_{2})+\lambda_{\mathrm{pixel}}\times \ell_{\mathrm{pixel}}\;(I_{1},\;I_{2})$$

The pixelwise loss (*ℓ_pixel_*) between pair of images ($I_{1},\;I_{2}$) is calculated as: (*ℓ_pixel_* ($I_{1},\;I_{2}$) = $\left\|I_{1}-I_{2}\right\|$). The adversarial loss (*ℓ_adv_*) between the generator and discriminator (*g*, *d*) is:

(3) $$\ell_{\mathrm{adv}}(g,d)=\min(\max(\sum \ell_{\mathrm{GAN}}(g,d))\;\ell_{\mathrm{GAN}}\;(g,\;d)=E_{\left(I_{1},I_{2}\right)}[\log\;d(I_{1},\;I_{2})]+E_{I_{1}}[\log(1-d(I_{1},\;g(I_{1})))]$$

Here, ‘$E_{\left(I_{1},I_{2}\right)}$’ is the expected value over all real data instances. ‘$E_{I_{1}}$’ is the expected value over all random inputs to the generator. The reenactment generator loss (*ℓ_RG_*) is given by Equation (4):

*ℓ_RG_* = *ℓ_perc_* + *ℓ_rec_* + *ℓ_adv_* (4)

The cross-entropy loss (*ℓ_CE_*) is defined as:

*ℓ_CE_* = −∑(*t_i_* × *log*(*p_i_*)) (5)

Here, ‘*t_i_*’ is the truth label, and ‘*p_i_*’ is the ‘*softmax*’ probability for the *i^th^* class. The loss of the segmentation generator (*ℓ_SG_*) is obtained by Equation (6):

*ℓ_SG_* = *ℓ_CE_* + *ℓ_pixel_* (6)

The loss of the in-painting generator (*ℓ_ip_*) and loss function (*ℓ_b_*) for facial blending are calculated in Equations (7) and (8), respectively:

*ℓ_ip_* = *ℓ_rec_* + *ℓ_adv_* (7)

*ℓ_b_* = *ℓ_perc_* + *ℓ_adv_* (8)

## 7. In-cabin Monitoring System (IMS)

**Figure 11:**
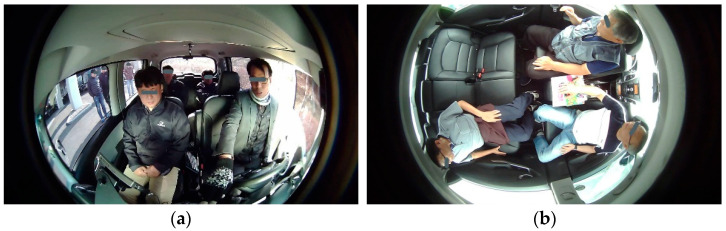
Example image representing the comparison of camera positioning: (**a**) FF monitoring camera; (**b**) The proposed camera installed at the center of the RT of AV. The FF camera captures both in-cabin and outside. The camera installed at the center of the RT of the AV captures only in-cabin. Faces are masked for privacy preservation.

We have proposed an intelligent monitoring system for the in-cabin monitoring of level 4 and beyond AVs. It consists of a set of intelligent monitoring cameras assisted with AI technologies. Figure 1 has depicted an important question about the positioning (installation) of the intelligent monitoring camera inside the cabin of the AV. Figure 11 presents a comparison of monitoring the in-cabin by installing the monitoring camera at different positions. The monitoring camera captures outside of the cabin along with in-cabin when installed in front-facing (FF). Therefore, the object detector algorithm misinterprets the person outside of the vehicle as to the occupant of the vehicle. However, such situations can easily be omitted through the rooftop (RT) camera installation. The camera at the center of the rooftop captures only the in-cabin of AV (depicted in Figure 11).

**Figure 12:**
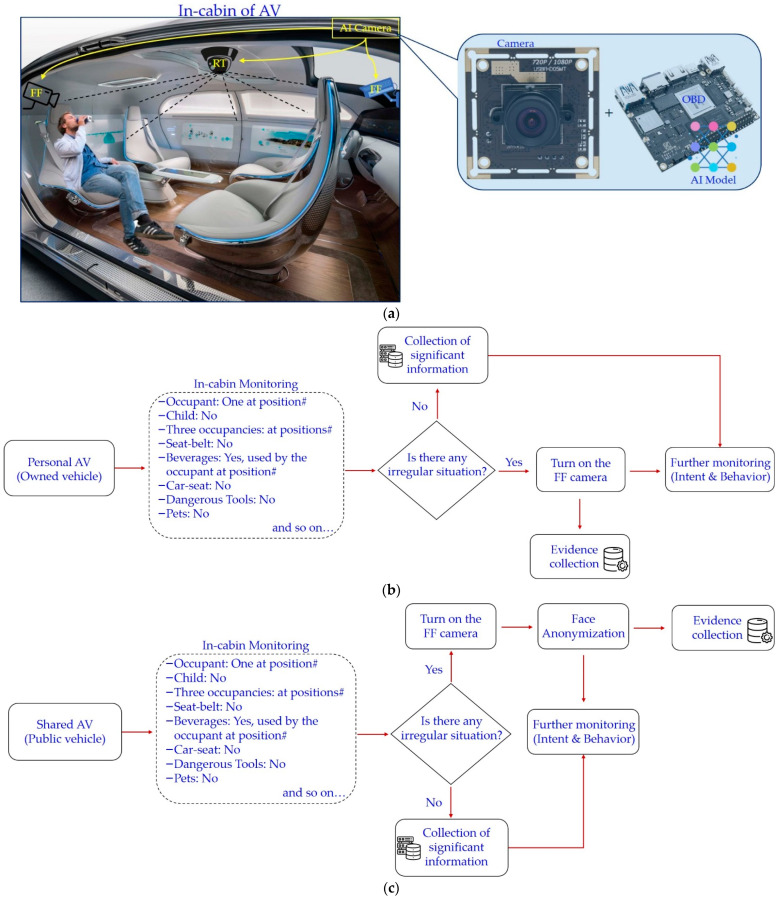
The proposed IMS for level 4 and beyond AVs: (**a**) Setup of proposed IMS; (**b**) IMS for personal AV (i.e., owned vehicle for personal use only); (**c**) IMS for shared AV (i.e., public vehicles or transport vehicles). Here, FF: Front-facing monitoring camera; RT: Rooftop monitoring camera.

Though the RT camera positioning supports the privacy of the occupants in some respect, it fails in the details of the faces. Facial information is important for intent, behavior, and other monitoring objectives. Therefore, the FF camera is utilized to detect the facial information of the occupant during monitoring of an irregular situation and for evidence collection. However, facial information leads to a breach in the individual’s privacy. Therefore, the FF camera becomes operational only in case there is any abnormal situation. Figure 12 renders the proposed IMS. It consists of an AI camera that has the capability of multifaceted in-cabin monitoring. The proposed IMS is an on-device AI-based OBD resembling a black box that provides inbuilt information security to the proposed system.

An AI camera is installed at the center of the vehicle such that the entire vehicle can be monitored in a single shot. It is equipped with a camera and an AI-assisted onboard device (OBD) to recognize objects and events. There is a set of front-facing cameras installed in the vehicle’s cabin for the collection of pieces of evidence and identification of the victims and culprits under an irregular situation. We have considered two types of IMSs depending on the AV ownership and its services. One is for a personal AV, and the other is for public AVs (shared and transport vehicles). In personal AVs, there is no information security threat because the proposed IMS is based on the on-device AI. There is no privacy threat in the on-device AI systems. Therefore, all the authorities accessing a piece of information belong to the owner of the vehicle. In contrast, public AVs have problems with privacy. To subdue that privacy breach, we have adopted facial anonymization in such IMSs. Both of these IMSs are shown below in Figure 12.

The proposed AI camera of the IMS has a CMOS camera and an OBD. The digital camera for the proposed IMS should be an image sensor (color) with a low voltage, high-performance, and full-size high-definition image capture quality (such as 1080 p, i.e., 1920 × 1080 pixel image resolution) omnidirectional image quality in a smaller package. Therefore, we have used the full HD 1080 P camera with Omnivision *OV2710* CMOS sensor (model no: CMT-2 MP-OV2710-R020-C) as the AI camera in our proposed IMS setup. It is capable of 1080 p (here, p is progressive scan, and 1080 represents 1920 × 1080 progressively displayed pixels, also known as Full HD (full high-definition)) recording with a high-speed 30 fps (frames per second) without any distortion lens. It captures the entire cabin of the AV in a single shot without any interference from the outer environment of the cabin. The proposed IMS has an AI-assisted OBD to recognize objects and events for mitigation of any irregular situation in-cabin of level 4 and beyond AVs. The AI camera supports color image sizes with 1080 p resolution at a frame rate of 30 fps and with 720 p resolution at a frame rate of 60 fps. It also supports video graphic array (VGA) resolution at the frame rate of 60 fps. It is based on CMOS technology. It is a lead-free image sensor with a 68-pin CSP3 chip-scale package type of integrated circuit. We have chosen it as an image sensor because the CMOS sensors use less power and provide faster readouts. They are also less vulnerable to static electricity discharges. The *Rockchip RK 3399* is used as an OBD in our proposed IMS. It has a six-core 64-bit server-level processor with a built-in neural processing unit (NPU) to support AI hardware acceleration. The power consumption of NPU for AI operation is less than that of a general-purpose graphical processing unit. In addition, it supports major in-depth learning models. Therefore, it is best suited as an OBD for AI applications.

Table 4 encapsulates the digital camera parameters of the considered AI camera in the proposed IMS.

## 8. Results and Discussion

The proposed IMS approach discerns the seamless monitoring of AV cabin for safety and security assurance. In this work, the following experiments have been performed:

- Determining the position of the AI camera;
- Comparison of popular AI models for object recognition for in-cabin monitoring;
- Object recognition for various scenarios to avoid any irregular situation.

### 8.1. AI Camera Positioning

We have performed an occupant detection experiment to show the efficacy of the proposed IMS approach and AI camera positioning.

**Figure 13:**
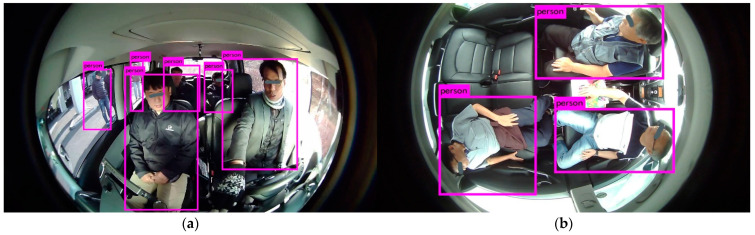
A comparison of occupant’s recognition using different positioning of monitoring camera: (**a**) Occupant’s recognition for FF camera; (**b**) Occupant’s recognition for RT camera.

Figure 13 showcase the difficulties and requirements of the in-cabin monitoring camera positioning. It is depicted in the figure that the camera installed front-facing has two critical issues for level 3 and level 4 AVs and two crucial issues in FAVs. In level 3 and level 4 AVs, front-facing installations suffer from outside (as demonstrated in Figure 13a. In addition, it leads to difficulty in monitoring the second row because of hindrances in the line of sight from the front row of the vehicle. FAVs have seating arrangements as depicted in Figure 13b. In such seating arrangements, two front-facing cameras will be required for complete monitoring of the in-cabin. Furthermore, it will double the same difficulty outside (as depicted in Figure 13a). The AI camera positioned anywhere else will have similar shortcomings. Therefore, the proposed position (i.e., RT center) is the best position for a robust IMS. The FF camera becomes operational when an abnormal situation occurs and there is a need for detailed monitoring or surveillance, as well as evidence collection.

### 8.2. Comparison of Popular Object Detection Models

**Figure 14:**
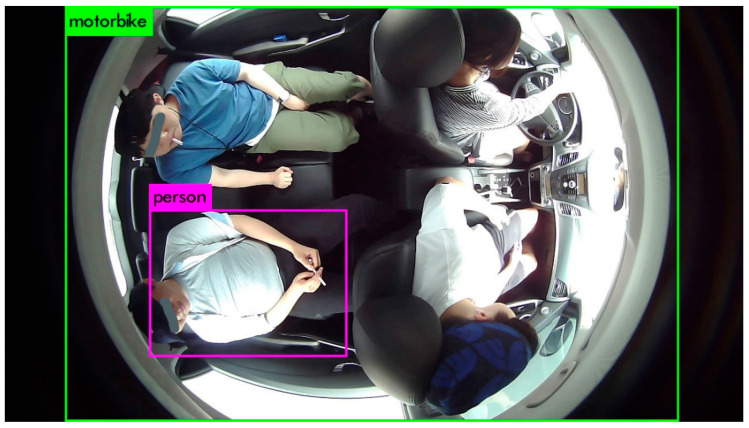
Direct inference through pretrained weights.

A comparison has been carried out considering popular AI algorithms to select the appropriate approach for object and occupant detection in the proposed IMS approach. Popular deep-learning-based algorithms in object detection are given and surveyed in [53,54,55,56,57,58,59,60]. Generally, they can be divided into two categories: a one-stage detector such as “you only look once (YOLO or yolo)” [53] and a two-stage detector such as a region-based convolutional neural network (R-CNN) [54]. Although there are different versions of both varieties, the effectiveness of the algorithms depends upon their application. One-stage algorithms are considered in a situation where latency in inference is concerned, whereas the two-stage detectors are found appropriate in terms of their accuracy. These algorithms are very commonly used in real-time object detection applications. YOLO utilizes nonmaximal suppression, whereas R-CNN uses a region-based feature extraction mechanism for object detection. The pretrained weights of both kinds of approaches are already available for plenty of object categories. However, direct usage of pretrained weights of these algorithms was producing incorrect inferences, as depicted in Figure 14. The viewing angle of the AI camera is the pertinent reason for this failure.

**Figure 15:**
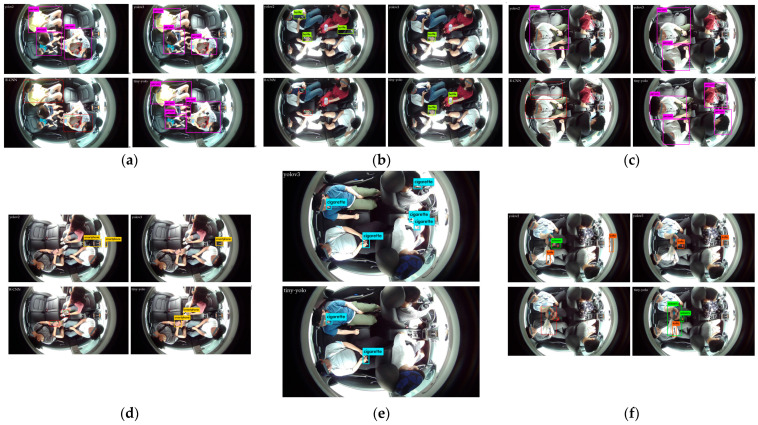
Performance comparison of object recognition using yolov2, yolov3, R-CNN, and tiny-yolo: (**a**) Occupancy; (**b**) Food and Beverages; (**c**) Occupants and Objects; (**d**) Cellular and Electronic Devices; (**e**) Smoking Items; (**f**) Harmful Objects.

Therefore, a fine-tuning of algorithms was necessary for our DB because of its top view instead of the front view. Various popular algorithms including YOLO versions (i.e., yolov2, yolov3, tiny-yolo), and R-CNN algorithms have been considered for comparison in our experiment. The comparison results are represented in Figure 15.

The performance comparison of various popular approaches, viz., yolov2, yolov3, R-CNN, and tiny-yolo, for object recognition is shown in Figure 15. We have chosen six different scenarios for the assessment of different object recognition approaches.

Table 5 presents the accuracy and inference time taken by the considered approaches. It elicits that tiny-yolo performs better in terms of average accuracy and inference time. Furthermore, in the considered frame in Figure 15e containing smoking items, the yolov2 and R-CNN have failed to produce an output. However, the tiny-yolo has correctly recognized the smoking items in the given scene. Therefore, we have chosen it for our proposed IMS. The accuracy comparison renders per frame predictions. As the images are samples from video frames where volunteers are allowed to perform the various tasks that are possible in the cabin of a vehicle. Hence, there is the possibility of a few blurry frames. Therefore, errors may occur during object recognition for a specific frame. However, overall object recognition should be considered on an average recognition performance basis. Therefore, the results obtained by the considered IMS using the tiny-yolo approach are all correct in each scenario.

### 8.3. In-Cabin Monitoring through Proposed IMS

**Figure 16:**
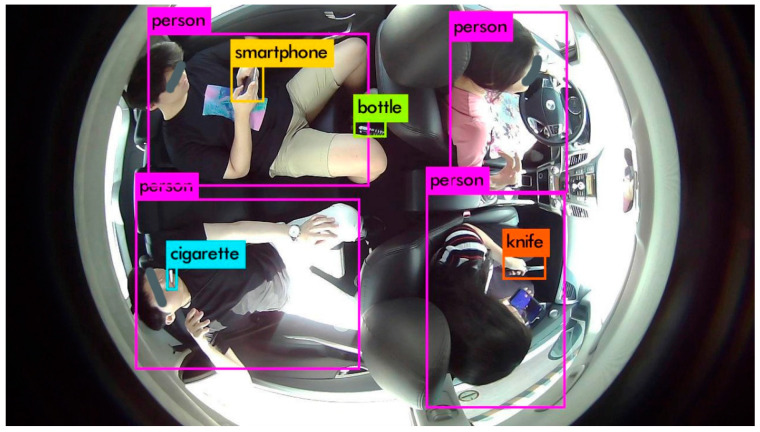
An illustration of in-cabin monitoring using the proposed IMS.

Using our generated DB containing real scenarios inside the cabin of a vehicle, we have applied our proposed IMS and monitored the cabin for various tasks. A few of the cases have already been depicted in the previous Figures. An illustration of the in-cabin monitoring using our proposed IMS is rendered in Figure 16.

The above results showcased that the suggested AI-camera-based IMS provides a robust solution for in-cabin monitoring to mitigate any irregular situation.

## 9. Discussion and Conclusions

Multi-pronged in-cabin monitoring is crucial in higher levels of autonomous driving to persuade the safety and security of both occupants and the vehicle’s in-cabin. Industries and academia are in demand of a robust IMS and a suitable voluminous DB. Our DB has incorporated a variety of possible scenarios including desired variations for in-cabin monitoring of AVs. In this study, we have considered both personal and shared AV scenarios. Our proposed IMS incorporates both the privacy and safety of occupants. The collected DB consists of a wide range of actions possible in an IMS to curb and mitigate an irregular situation. We focused on the issues for mobility services in autonomous driving. It requires uninterrupted and robust monitoring of the in-cabin to avoid an abnormal situation that can ensure the safety and security of the users and vehicle. 

In this view, our proposed system provides two-level monitoring (represented in Figure 12). In the first level, a normal monitoring task is performed using an RT camera, whereas second-level monitoring is needed to track the victim and culprit and to collect the shreds of evidence. In the case of personal AVs, privacy is not a major concern; however, it is of utmost importance in public AVs. However, the proposed IMS is based on on-device AI that has the inherent benefits of information security. Moreover, there is the requirement of merely trivial information sharing during the commute, such as the number of occupants, occupancy, etc. Privacy threats occurring during an abnormal situation are detected. We have advised facial anonymization to preserve individuals’ privacy.

Therefore, our proposed IMS provides a versatile solution required for in-cabin monitoring. It can be applied to lower as well as higher levels of autonomous driving. In level 2 and level 3, it can be considered as the assistance system. It fulfills the essential demand of safety and security of occupants for Level 4 AVs. In level 5 AVs, it will be an extension of the operational design domain (ODD).

Moreover, the proposed IMS is a robust solution for future intelligent vehicles. It is not possible to regulate the proposed AI cameras. They are the component of the OBD. Any unwanted activity against the OBD or the vehicle itself sets off alarms and is reported as unauthorized access. There is an option in recent vehicles to put their dashboard camera always in ON mode. It works as a monitoring device even during parking or in idle conditions to protect against any theft or tampering activity with the vehicle. In the working condition of the proposed IMS, it will record each and every activity of the occupants. Therefore, any kind of tampering or regulation with the camera will be detected, recorded, and reposted to the authorities and service providers.

**Figure 17:**
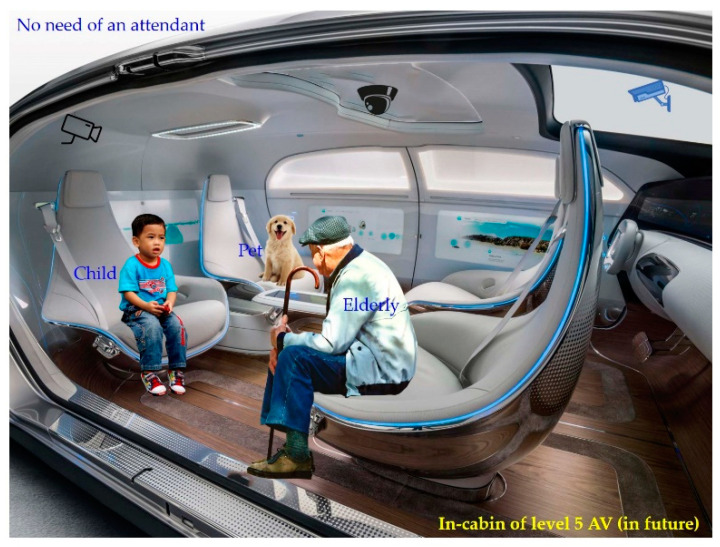
Example scenarios and use cases for an extension of the ODD of FAVs.

In the future, it will serve the demand for safety for those who currently require some sort of attendant with them in their commute, such as young children, disabled people, elderly people, etc. Even pets can be safely transported alone from one place to another. Figure 17 collectively represents some of such scenarios. The proposed monitoring system will help in legalizing the travel of such occupants alone in a vehicle because the proposed IMS ensures their safety via distant but robust monitoring. Moreover, the collected DB is available on demand for further research in this domain.

The online learning mechanism requires efficient hardware capable of heavy computations to support training as well. Currently, we have separately trained the model on our database and used the best-trained model for inferences. Future research in the relevant area may include online learning. It will facilitate the inference mechanism on the unseen environment as well. Popular techniques such as active learning and federated learning can be used to implement an online learning process for an unseen scenario. However, efficient hardware (e.g., AI accelerator) is essentially required to accomplish the online learning experiments. In addition, energy-efficient hardware is also needed to maintain the always ON mode of the monitoring camera and the IMS. Nevertheless, the proposed IMS serves as an efficient platform to accomplish all necessary in-cabin monitoring tasks of future intelligent vehicles.

## Figures and Tables

**Table 1:** The safety and security of the occupants and vehicle in the in-cabin monitoring of AVs.

Occupant		Vehicle Safety
Safety	Security	
Occupancy; No. of occupants; Age group, gender, etc.; Occupant body part outside the vehicle; Seat belt; Child age, car-seat, an unattended child, etc.; Eating, smoking, drinking, etc.; Spilled objects and belongings; Pets/Animals; Irregular situations. ^1^	Vandalism, teasing, abuse, argument, etc.; Dangerous objects, weapons, etc.; Personal privacy; Stealing, theft, etc.; Illegal activities; Irregular situations.	Misuse, mishandling, or malicious activities toward the vehicle; Irregular situations.

^1^ Irregular situation: A situation contrary to the normal rules or the established situation.

**Table 2:** Summary of the objectives and activities performed in our DB.

Objective	Behavior
Occupancy detection	No. of occupant seats
Sleep detection	Sleeping occupant
Seat belt detection	Person without a seat belt
Child, car-seat, an unattended child detection	A child without a car seat, an unattended child
Cellphone detection	Cellphone in use or unused
Food detection	Food and beverages, water bottle, in-use or unused
Harmful object detection	Tools, knife, scissors, harmful objects in hand
Smoking detection	Cigarette, E-cigarette, lighter
Objects detection	Belongings, bags, paper, etc.
Animal (pets) detection	Unattended animal (pets) detection

**Table 3:** Different targets with details and their number of images in our DB.

Target	Details	No. of Frames
Occupants and Objects	Adults, children, pets, car seat, seat belt, bags, belongings, etc.	148,878
Cellular and Electronic Devices	Laptops, smartphones, tablets, etc.	80,369
Smoking Items	Cigarettes, e-cigarettes, cigarette cases, and cigarette lighters	47,097
Foods and Beverages	Edibles, water, beverages, etc.	42,891
Harmful Objects	Tools, weapons, knives, scissors, etc.	13,865

**Table 4:** Parameters of AI camera (Omnivision OV2710 CMOS) used in the proposed IMS.

Parameter	Feature	
Model No	CMT-2MP-OV2710-R020-C	
Sensor	1/2.7″ Omnivision OV2710	
Pixel	2 Mega Pixel	
Most effective pixels	1920 (H) × 1080 (V)	
Pixel Size	3.0 µm × 3.0 µm	
Image area	5856 µm × 3276 µm	
Object distance	5 CM–100 M	
Output Parameter	Compression format	MJPEG/YUV2 (YUYV)
	Resolution	1080 P
Frame rate	30 fps	
Shutter Type	Electronic rolling shutter	
Focus type	Fixed focus	
S/N ratio	39 dB	
Dynamic range	69 dB	
Sensitivity	3300 mV/Lux-sec	
Adjustable parameter	Brightness/Contrast/Color saturation/Hue/Definition/ Gamma/White balance/Exposure	
Lens	Focal length: 3.6 mm Lens Size: 1/2.5 inch FOV: 90° Thread Size: M12 × P0.5 (Thread diameter is 12 mm, and pitch is 0.5 mm)	
Audio frequency	Optional	
Power supply	USB BUS POWER Analog: 3.0~3.6 V (3.3 V typical) Core: 1.425~1.575 V (1.5 V typical) I/O: 1.7~3.6 V (1.8 V typical)	
Power consumption	DC 5 V, 150 mW–200 mW	
Main chip	DSP/SENSOR/FLASH	
Support	Auto Exposure Control (AEC); Auto White Balance (AEB); Auto Gain Control (AGC)	
Storage temperature	−30 °C to 70 °C	
Operating temperature	0 °C to 60 °C	
OS support	WinXP/Vista/Win7/Win8/Win10	
Connectivity	USB video class (UVC) complaint, Plug and Play	

**Table 5:** A comparison of accuracy and inference time by different approaches.

Object Recognition Approach	Accuracy	Inference Time (in Sec.)
yolov2	84.54%	0.994
yolov3	87.64%	1193
R-CNN	87.74%	1.989
tiny-yolo	96.03%	0.163

## Data Availability

The required image data used to support the findings of this study are included in this article. The complete database is available on request from the corresponding author.
